# The Effects of Lactose Induction on a Plasmid-Free *E. coli* T7 Expression System

**DOI:** 10.3390/bioengineering7010008

**Published:** 2020-01-06

**Authors:** Johanna Hausjell, Regina Kutscha, Jeannine D. Gesson, Daniela Reinisch, Oliver Spadiut

**Affiliations:** 1 TU Wien, Institute of Chemical, Environmental and Bioscience Engineering, Research Division Biochemical Engineering, 1060 Vienna, Austria; johanna.hausjell@tuwien.ac.at (J.H.); regina_phf1@yahoo.de (R.K.); 2 Boehringer Ingelheim RCV GmbH & Co KG, 1120 Vienna, Austria; jeannine.gesson@boehringer-ingelheim.com (J.D.G.); daniela.reinisch@boehringer-ingelheim.com (D.R.)

**Keywords:** *E. coli*, T7 expression system, plasmid-free expression, lactose induction, antigen binding fragment (Fab), lac-repressor (LacI)

## Abstract

Recombinant production of pharmaceutical proteins like antigen binding fragments (Fabs) in the commonly-used production host *Escherichia coli* presents several challenges. The predominantly-used plasmid-based expression systems exhibit the drawback of either excessive plasmid amplification or plasmid loss over prolonged cultivations. To improve production, efforts are made to establish plasmid-free expression, ensuring more stable process conditions. Another strategy to stabilize production processes is lactose induction, leading to increased soluble product formation and cell fitness, as shown in several studies performed with plasmid-based expression systems. Within this study we wanted to investigate lactose induction for a strain with a genome-integrated gene of interest for the first time. We found unusually high specific lactose uptake rates, which we could attribute to the low levels of lac-repressor protein that is usually encoded not only on the genome but additionally on pET plasmids. We further show that these unusually high lactose uptake rates are toxic to the cells, leading to increased cell leakiness and lysis. Finally, we demonstrate that in contrast to plasmid-based T7 expression systems, IPTG induction is beneficial for genome-integrated T7 expression systems concerning cell fitness and productivity.

## 1. Introduction

*E. coli* is one of the most widely used hosts for recombinant protein production to date. Genetic manipulation is easy and versatile, there are numerous strains and plasmids available, and the cells can be cultivated fast, in cheap media, to high cell densities [1,2,3,4,5].

Conventionally, plasmids are used for recombinant protein production in *E. coli*, including the most frequently employed pET vectors, commercially established by Novagen^TM^. On pET plasmids, the gene of interest (GOI) is encoded under control of the T7 promoter, which is recognized by T7 RNA polymerase. Compatible strains like *E. coli* BL21 (DE3), have the λDE3 region integrated into the genome, where transcription of T7 RNA polymerase is controlled by the *lac*UV5 promoter. Therefore, transcription and translation of the GOI depends upon transcription and translation of the T7 RNA polymerase from the *lac*UV5 promoter. Before induction, transcription of the T7 RNA polymerase and consequently the GOI are inhibited by the lac-repressor (LacI) which binds in a tetrameric structure to the respective operator sites [6]. *Lac* operator sites are located in (I) the native *E. coli lac* promoter (II) the *lac*UV5 promoter, which regulates transcription of the T7 RNA polymerase and (III) in the *lac* operator regions included downstream of the T7 promoter and upstream of the translation initiation sequence of the GOI for ensuring tighter transcription control [6]. A copy of *lacI* is present in the genomic DNA of the cell as well as on pET plasmids. During induction, the LacI-tetramer is bound by allolactose or one of its analogs, dissociates from the operator-sites and enables transcription and translation of the T7 RNA polymerase and thus in turn expression of the GOI from the T7 promoter [7,8,9].

Unfortunately, plasmid-based expression systems exhibit certain drawbacks. They either (I) tend to amplify the plasmid copy number in prolonged cultivations, or (II) plasmids are lost over time, propagating the segregation of a plasmid-free sub-population during induction. The latter has also been described in context with T7-pET systems, making establishment of stable production processes challenging [10,11].

One way to overcome these challenges is employment of plasmid-free expression systems, where the GOI is integrated directly into the host genome. A number of recombination methods have been developed for establishment of such systems and different suitable chromosomal integration sites in *E. coli* have been investigated [12,13]. Once the GOI is integrated into the genome, recombinant production is no longer subject to plasmid number variations, allowing, aside from stable processes, the establishment of a reference for the performance of plasmid-based systems as well. However, one of the major drawbacks of moving away from plasmid-based systems remains, leading to a slightly limited production capacity due to the lowered copy number of the GOI [1,14,15,16].

The gold standard for induction in T7 expression systems is IPTG, as it ensures stable and strong induction since it is not metabolized by the cells. This makes one point addition sufficient, easing handling of bioprocesses. Nevertheless, it has been reported that IPTG puts a high metabolic burden on the cells, decreases the amount of soluble recombinant protein, and exacerbates substrate toxicity [17,18,19]. One way to tackle these adverse effects is to use lactose as inducer, which has been shown to yield in similar if not higher product titers and to increase soluble product formation and cell fitness, enabling longer production times [19,20,21,22]. Additionally, lactose is cheap and non-toxic. Nevertheless, it has to be kept in mind that the disaccharide has to be supplied constantly, as it is rapidly metabolized by the cells.

Recent studies from our group showed that there is a correlation between the maximum specific lactose uptake rate (q_s,lac,max_) and the specific glucose uptake rate (q_s,glu_) in *E. coli* BL21 (DE3) strains carrying pET plasmids. For several different pET plasmids for expression of various products, a mechanistic model for this correlation has been established, which can serve as a basis for steering product titers, product properties and/or product location [20,23,24].

Within this study we wanted to investigate the potential differences in the correlation between q_s,glu_ and q_s,lac,max_ for a strain where the GOI was not located on a pET plasmid but genome-integrated, knowledge which has not been generated to date. We wanted to shed light on the role of the plasmid and investigate if differences in the correlation were detectable when switching from plasmid-based to genome-integrated systems. As a model protein FabZ, an antigen binding fragment (Fab), was used which was translocated into the periplasm, as the oxidizing environment there allows necessary formation of disulfide bonds. As it is well known that periplasmic protein production can lead to cell leakiness and lysis [25], we further investigated if lactose induction had a positive effect in this regard. Finally, we compared induction by lactose to a standard IPTG-induced FabZ production process concerning productivity as well as physiology and viability.

## 2. Materials and Methods

### 2.1. Strains

In this study we used an *E. coli* BL21 (DE3) strain, which was provided by Boehringer Ingelheim RCV. The genes for the FabZ heavy chain and the FabZ light chain were encoded under the control of the T7 promoter and integrated into the genome at the attTN7 site by recombineering according to [26]. The homologous overhangs were each approximately 50 bp. Each gene was preceded by an *omp*A leader sequence (signal sequence for translocation into the periplasm) and a ribosomal binding site for efficient translation. A bicistronic mRNA was used, were the T7 promoter is followed by the open reading frame of the light chain including the *omp*A leader sequence and subsequently the open reading frame of the heavy chain also including the *omp*A leader sequence. Additionally, the strain carried a kanamycin-resistance. The ribosome binding site used was aagaaggaga. The *ompA*-leader sequence was MKKTAIAIAVALAGFATVAQA in terms of amino acids, the respective DNA-sequence was optimized together with the light and heavy chain of FabZ. Sequence details of the product FabZ are confidential. The heavy chain is 23.2 kDa and has a pI between 9.05 and 9.06 (depending on the prediction tools used), the light chain is 23.1 kDa and has a pI between 5.62 and 5.74. The full construct is 46.3 kDa and has a pI of 8.41. The overall integration cassette consisted of the T7-promoter, the ribosome binding site, *ompA*-light chain, ribosome binding site, *ompA*-heavy chain, the T7-terminator and a kanamycin resistance gene. Regarding the promoter, terminator, *ompA* and the kanamycin resistance gene, standard sequences were used.

To study the influence of the lac-repressor (LacI), the strain was transformed with an empty pET-21 d (+) plasmid in the course of our investigation.

### 2.2. Transformation with pET-21 d (+)

A heat-shock transformation of *E. coli* BL21(DE3) with pET-21 d (+) was performed. In order to produce chemically competent cells, the cells were grown in LB-medium at 37 °C and 230 rpm to an optical density of about 0.5 at 600 nm. After centrifugation (2500× *g*, 4 °C, 10 min) the supernatant was discarded and the cells were resuspended in 2 mL of ice-cold 30 mM CaCl_2_. Subsequently, the cells were spun down for 30 s and the pellet was gently resuspended in 0.5 mL of ice-cold 30 mM CaCl_2_.

50 µL of this cell-suspension were mixed with 3 µL (442 ng) of plasmid DNA and incubated on ice for 30 min. Then the mixture was incubated at 42 °C for 30 s and then on ice for 5 min. Next, 950 µL of SOC-medium (0.5% yeast extract, 2% tryptone, 10 mM NaCl, 2.5 mM KCl, 10 mM MgCl_2_, 10 mM MgSO_4_, and 20 mM glucose) were added, and the cells were regenerated at 30 °C and 180 rpm for 1 h.

For selection, the cells were grown on LB-agar plates containing 50 µg/mL kanamycin and 100 µg/mL ampicillin at 30 °C for 72 h. Successful transformation of the cells with the plasmid was confirmed by isolation and commercial sequencing (Microsynth Austria AG, Vienna, Austria).

### 2.3. Bioreactor Cultivations

Cultivations were carried out once in the controlled environment of a bioreactor, closing C-balances were used as a control for calculations. Measurements and analyses of samples were carried out in triplicates. All bioreactor cultivations included a batch phase, an uninduced fed-batch phase to a dry cell weight concentration of 30 g/L, and an induction phase of 6 h.

Cultivations were performed in DASbox^®^ Mini Bioreactors (Eppendorf, Hamburg, Germany) with 250 mL working volume. The pH was measured via pH-Sensor EasyFerm Plus (Hamilton, Reno, NV, USA) probes, dissolved oxygen with Visiferm DO 120 electrodes (Hamilton, Reno, NV, USA), and CO_2_ and O_2_ in the offgas via a DASGIP^®^ GA gas analyzer (Eppendorf, Hamburg, Germany). For aeration a mixture of pressurized air and pure oxygen was provided at 18 L/h. The stirring speed was kept at 2000 rpm. The dissolved oxygen saturation was held above 30% by adding more pure oxygen if required. The feed-flowrates were regulated via a DASbox^®^ MP8 Multi Pump Module, the pH was kept at 6.8 during batch and fed-batch phase, and was adjusted to 7.1 during the induction phase. The temperature was controlled at 37 °C during the growth phases and subsequently lowered to 32 °C for the induction phase. The process parameters were recorded and controlled by DASware^®^ control.

Five hundred milliliters of DeLisa pre-culture medium [27], containing 30 mg/L kanamycin, were inoculated aseptically with one frozen stock of cells (−80 °C; 1.5 mL) and incubated overnight (approximately ~17 h) in 2500 mL high-yield shake flasks at 37 °C and 200 rpm. The DeLisa batch medium was inoculated with 10% of the starting volume. After the sugar was depleted, indicated by a drop in the CO_2_ signal, the biomass concentration was further increased (to approximately 30 g/L) by carrying out a fed-batch phase with a glucose feed of 400 g/L. The production of FabZ was either induced with 1.25 mM IPTG, as used the industrially developed process, following an induction phase with an average q_s,glu_ of 0.13 g/g/h, or, for lactose induction, two feeds including a 400 g/L glucose feed as well as a 200 g/L lactose feed were utilized to achieve the desired specific sugar uptake rates.

### 2.4. Feed Control Strategy

The amount of feed added to the reactors for exponential fed-batch phases was calculated by the control software using a simple feed-forward exponential feeding strategy, according to the following equation:

(1) $$Feed\;setpoint\;\left[\frac{mL}{h}\right]=q_{s}*V*c_{x}*e^{\left(µ\;*t\right)}*\frac{1000}{c_{s}}.$$

µ specific growth rate on glucose/lactose [h^−1^]
q_s_ specific glucose/lactose uptake rate [g_x_/g_s_/h]
*V* reactor volume [L]
*c_x_* biomass concentration [g_x_/L]
*t* time [h]
*c_s_* concentration of the feed [g_s_/L]

The growth rate was calculated from the specific sugar uptake rates and yields on the respective sugars.

### 2.5. Sampling

Samples were taken at the start of the batch phase, at the start of the fed-batch phase, at the beginning of the induction, and in different intervals during induction. For cultivations needed for the correlation between q_s,glu_ and q_s,lac,max_ the sampling interval was one hour, for further experiments concerning tunability and the influence of LacI the sampling interval was widened to 6 h. For every sample the optical densities at 600 nm (OD_600_) and at 550 nm (OD_550_) were determined spectroscopically using a Genesys 20 photometer (Thermo Scientific, Waltham, MA, USA), using appropriate dilutions to stay in the linear range (OD_600_ 0.2–0.8).

Biomass aliquots of each sample were generated by pipetting an amount of cell broth calculated via Equation (2),

(2) $$sample\;volume\;\left[mL\right]=\frac{10}{OD_{550}},$$

into 500 µL of 0.9% NaCl solution.

These aliquots were then centrifuged at 20,000× *g* at 4 °C for 10 min in a Sigma 3-18K centrifuge. The supernatant was discarded and the samples were frozen at −20 °C for product analytics.

Dry cell weight was determined gravimetrically by pipetting 1 mL of sample into pre-weighed 2 mL Eppendorf tubes, centrifuging at 20,000× *g* at 4 °C for 10 min using a Sigma 3-18K centrifuge (Sigma Laborzentrifugen GmbH, Osterode am Harz, Germany), resuspending the pellet in 1 mL of 0.9% NaCl, centrifuging again at the same conditions and drying the pellets at 110 °C for 72 h. Subsequently, the pellets were weighed.

Additionally, at the end of the cultivation, several 10 mL samples of culture broth were taken, centrifuged at 5000× *g* for 30 min at 4 °C and the pellet was frozen at −20 °C for later homogenization.

The concentrations of glucose, lactose, galactose, and acetate were determined via HPLC in the cell-free supernatant (Thermo Fisher Scientific, Waltham, MA USA) with an Aminex HPX-87H Column (Bio-Rad, Hercules, CA, USA) using 4 mM H_2_SO_4_ for isocratic separation and a RefractoMax520 refractive index detector (DataApex, Petrzilkova, Prague, Czech Republic). The flowrate was set to 0.6 mL/min and the method lasted for 30 min. All analyses were performed in triplicates.

### 2.6. Product Analytics

Product analytics of soluble product in the cells and in the cell-free supernatant were carried out according to procedures previously described in literature [28].

### 2.7. Leakiness (Alkaline Phosphatase Assay)

Within our study the term “leakiness” refers to cells where alkaline phosphatase and FabZ can be detected in the culture supernatant, indicating a permeable outer membrane. This includes cells, which are lysed and have an open outer membrane for this reason. However, we additionally determined DNA contents in the culture supernatant, which rise upon cell lysis. To determine cell leakiness, an alkaline phosphatase assay was performed with the cell-free supernatants according to literature [25]. For comparability reasons between different cultivations, the slopes of the measurements were divided by the biomass concentration.

Additionally, cells were homogenized by resuspending the pellets from the 10 mL samples in buffer A (20 mM NaH_2_PO_4_·2H_2_O, 100 mM NaCl, pH 7) and running 10 passages at approximately 1000 bar in a Panda Plus Homogenizer (GEA Niro Soavi, Parma, Italy). After the subsequent centrifugation at 15,000× *g* and 4 °C for 20 min, alkaline phosphatase activity was measured in the supernatant. The slopes of these measurements were again normalized by biomass used for homogenization. The resulting values were defined as 100% leakiness for the different cultivations.

### 2.8. Lysis (Picogreen Assay)

To estimate the amount of lysed cells, Quant-iT™ PicoGreen^®^ dsDNA assays (Invitrogen™, Thermo Fisher Scientific, Waltham, MA USA) were performed using a Quant-iT™ PicoGreen™ dsDNA Assay Kit. After preparing a working solution (200-fold dilution of the concentrated DMSO solution of Quant-iT™ PicoGreen^®^ reagent in TE-buffer (10 mM Tris-HCl, 1 mM EDTA, pH 7.5)), 100 µL thereof were mixed with 100 µL of 100-fold diluted cell-free supernatant and incubated in the dark at room temperature for 5 min. Fluorescence was measured by exciting at 480 nm and measuring the emission at 520 nm using an Infinite 200 Pro plate reader (Tecan, Zürich, Switzerland). The calibration was done with the lambda DNA standard provided in the assay kit in a range of 1 ng/mL to 1 µg/mL. In order to compare the results of different cultivations at different times, the resulting DNA-concentrations were normalized by biomass.

## 3. Results and Discussion

### 3.1. Correlation between q_s,glu_ and q_s,lac,max_

**Figure 1:**
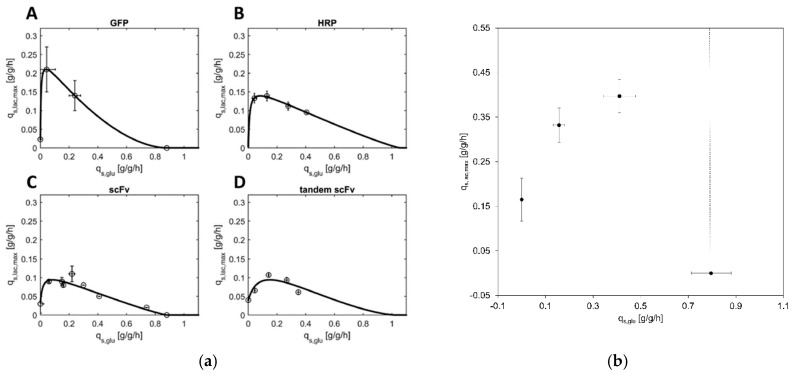
q_s,glu_/q_s,lac,max_-correlations (**a**) q_s,glu_/q_s,lac,max_-correlation for recombinant pET-based *E. coli* BL21 (DE3) strains producing either (A) GFP, (B) HRP, (C) the scFv or (D) the tandem scFv. Data-points were obtained from batch and fed-batch cultivations with constant q_s,glu_ and excess lactose (“static experiments”) and subsequently fitted to the mechanistic model (Equation (1)) reprinted from [23] as a comparison to previously recorded correlations between q_s,glu_ and q_s,lac,max_ for plasmid-containing BL21 (DE3) strains. (**b**) q_s,lac,max_ as a function of q_s,glu_ for the GI strain; the data-points were gained from the different batch and fed-batch cultivations: Point 1 (q_s,lac,max_ 0.17 ± 0.05 g/g/h at q_s,glu_ 0 g/g/h) is derived from a fed-batch only on lactose, the two middle points (q_s,lac,max_ 0.33 ± 0.05 g/g/h at q_s,glu_ 0.19 ± 0.04 g/g/h; q_s,lac,max_ 0.40 ± 0.04 g/g/h at q_s,glu_ 0.41 ± 0.07 g/g/h) were calculated from fed-batch cultivations where limiting amounts of glucose were fed and lactose was provided in excess, and point 4 (on the right) indicates the value of the maximum specific glucose uptake rate. Above this point, also indicated by a dotted line, glucose would accumulate and therefore no lactose uptake would occur due to the well-known phenomenon of diauxic growth and carbon catabolite repression whenever glucose and lactose are present in excess e.g., [29,30,31]. The error bars indicate the standard deviation of q_s,glu_ and q_s,lac,max_ during the cultivations.

We wanted to shed light on the mechanistic correlation between the specific glucose uptake rate (q_s,glu_) and the maximum specific lactose uptake rate (q_s,lac,max_) for a genome-integrated FabZ *E. coli* BL21 (DE3) strain (hereafter referred to as GI-strain). Previously, this correlation had only been recorded for plasmid-based T7 expression systems, where results showed that the correlation differed, dependent on the pET-plasmid and the expressed protein (Figure 1a) [23].

To determine the correlation between q_s,glu_ and q_s,lac,max_ for the GI strain, bioreactor cultivations at different q_s,glu_ set-points were carried out while supplying lactose in excess. The excess lactose supply was confirmed by HPLC-measurements, where lactose was detected in the supernatant of the culture broth at quantities greater than 0.5 g/L at all times. The actual specific lactose uptake rate was determined after an adaption phase ranging from 2 h to 5 h, which depended on the specific glucose uptake rate. The end of the adaption phase was indicated by a subsequently constant q_s,lac,max_.

Based on previously recorded q_s,glu_-q_s,lac,max_ correlations for plasmid-carrying *E. coli* BL21 (DE3) [23], we expected very little lactose uptake (q_s,lac,max_ below 0.05 g/g/h) in the complete absence of glucose. Additionally, we expected the highest q_s,lac,max_ at a specific glucose uptake rate of about 0.1 to 0.2 g/g/h. At those values usually q_s,lac,max_ of 0.1 to 0.2 g/g/h were reached (Figure 1a).

However, as shown in Figure 1b, the correlation for the GI strain differed strongly. In contrast to plasmid-based systems, where we detected almost no capability for lactose uptake if glucose was absent (q_s,lac,max_ < 0.05 g/g/h), we noticed considerable cell growth (Supplementary Table S1) and lactose uptake in the GI strain without supplementation of glucose (q_s,lac,max_ = 0.17 g/g/h).

**Figure 2:**
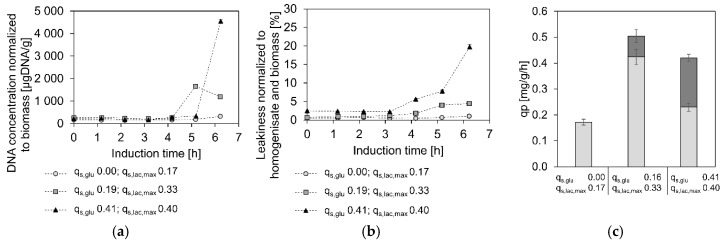
Leakiness, lysis and productivity at different q_s,glu_/q_s,lac,max_ (**a**) Lysis normalized to biomass at the different q_s,glu_ and correlating q_s,lac,max_ values: black triangles indicate values for q_s,glu_ = 0.41 g/g/h q_s,lac,max_ = 0.40 g/g/h, grey squares represent q_s,glu_ = 0.19 g/g/h q_s,lac,max_ = 0.33 g/g/h, light grey circles are values obtained from the lactose batch experiment. (**b**) Leakiness normalized to biomass: Black triangles indicate values for q_s,glu_ = 0.41 g/g/h q_s,lac,max_= 0.40 g/g/h, grey squares represent q_s,glu_ = 0.19 g/g/h q_s,lac,max_ = 0.33 g/g/h, light grey circles are values obtained from the lactose batch experiment. (**c**) specific overall, intracellular (light grey), and extracellular (dark grey) product formation rates for the q_s,glu_ and correlating q_s,lac,max_ values.

Aside from this astonishingly high lactose uptake in the absence of glucose, we also observed differences in the maximum of the q_s,glu_–q_s,lac,max_ correlation. With 0.40 g/g/h, the highest q_s,lac,max_ of the GI strain was almost double the amount of the highest previously recorded q_s,lac.max_, which was just above 0.2 g/g/h for an *E. coli* BL21 (DE3) strain, expressing green fluorescent protein from a pET-21 a (+) plasmid. Further, the highest q_s,lac,max_ of the GI strain occurred at clearly elevated q_s,glu_-values. While in plasmid-based systems the highest q_s,lac,max_ correlates to q_s,glu_ values between 0.1 and 0.2 g/g/h, the highest q_s,lac,max_ recorded for the GI strain is found at a q_s,glu_ of 0.41 g/g/h. Unfortunately, we were not able to determine q_s,lac,max_-values at higher q_s,glu_, since the cells tended to get leaky and lyse very quickly (in a matter of about 6 h; Figure 2a) if exposed to excess lactose for a prolonged time. Consequently, the cells had barely any time to fully adapt to lactose before lysing and there was no possibility to analyze the correlation at higher q_s,glu_ points, rendering a mechanistic modelling, as it has been done before [23], impossible.

Nevertheless, we investigated productivity at the different points along the q_s,lac,max_–q_s,glu_ correlation curve. As shown in Figure 2c, the highest overall specific soluble product formation rate (q_p_) was detected at a medium q_s,glu_ of 0.19 g/g/h and a q_s,lac,max_ of 0.33 g/g/h. The cultivation at a q_s,glu_ of 0.41 g/g/h and a q_s,lac,max_ of 0.40 g/g/h yielded less FabZ per biomass and hour and the lactose batch cultivation only exhibited a q_p_ below 0.2 mg/g/h. The overall trend, that q_p_ is lower when little glucose is taken up but also when q_s,glu_ is high, is the same, as previously found for plasmid-based systems [24], although it has to be kept in mind, that only soluble product was investigated in this study.

Interestingly, the q_p_ loss due to extracellular product clearly increased with higher specific lactose and glucose uptake rates. This most likely results from cell leakiness and lysis, as both seem to increase similarly at higher sugar uptake rates (Figure 2a,b). We hypothesized that at high specific lactose uptake rates the so-called lactose killing was triggered [32]. This phenomenon describes the death of *E. coli* grown on excess lactose and has been attributed to the transport of lactose through the cell membrane, influencing the proton motif force and leading to cell death [33]. In our case, the high specific glucose uptake rates and product formation in the periplasm might have additionally promoted cell lysis.

Summing up, we found similar trends for the productivity of the GI strain and plasmid-based expression systems along the q_s,lac,max_–q_s,glu_ correlation curve, however, the curve of the GI strain itself was clearly higher and its maximum was shifted to the right. Previous cultivations for the plasmid based expression systems were carried out at 30 °C, however, we chose 32 °C for the induction of FabZ expression, as this was determined as optimal induction temperature for this protein. Although sugar uptake rates and growth rates rise with higher temperatures, we found it rather unlikely that rising the temperature by 2 °C was the cause for almost doubling the lactose uptake rates.

Instead, we hypothesized that the reason for this strong divergence could originate from the plasmids. All employed pET plasmids from our previous studies [20,23,24,34] were high copy number plasmids, which also carried a *lacI* gene, thereby introducing much more repressor protein into the cells. The GI strain however, only expressed LacI from the copies in the genome [35]. Consequently, in the GI strain, less LacI would bind to the *lac* promoter and expression of lactose permease was elevated, allowing higher specific lactose uptake rates.

### 3.2. The Influence of LacI

**Figure 3:**
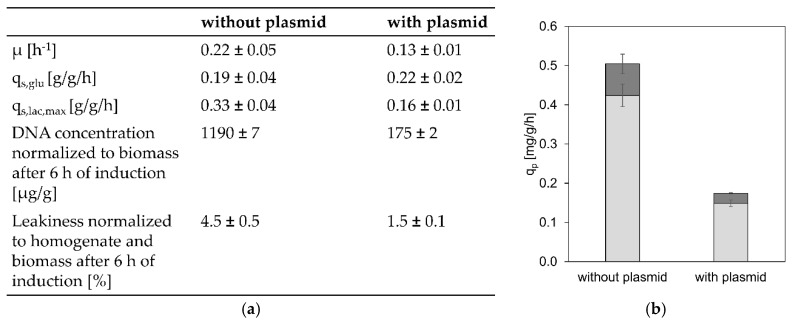
Physiology, leakiness, lysis and productivity for the GI-strain with and without an additional pET plasmid. (**a**) Comparison of physiological data as well as leakiness and lysis of a cultivation of the GI strain without plasmid and the GI strain containing the empty pET-21 d (+) plasmid; (**b**) Specific overall, intracellular (light grey), and extracellular (dark grey) product formation rates compared between the cultivations with and without plasmid.

To test our hypothesis that the GI strain exhibited higher q_s,lac,max_ values compared to plasmid-based systems, as a result of less *lacI* copies, an empty pET-21 d (+) plasmid was introduced into the GI strain. The high copy number plasmid carried an additional *lacI* gene. The transformed cells were cultivated in a bioreactor under the same conditions as before with an excess of lactose, at a q_s,glu_ comparable to 0.19 g/g/h. Specific sugar uptake rates, productivity, and leakiness/lysis were evaluated for the plasmid containing GI strain and compared to previous results of the GI strain without plasmid (Figure 3).

At a comparable q_s,glu_, the plasmid-containing strain exhibited only a q_s,lac,max_ of 0.16 g/g/h, approximately half of that of the GI strain without the plasmid and in the range of previously recorded q_s,lac,max_ values, which were found between 0.1 and 0.2 g/g/h. This confirmed our hypothesis that the shift in the q_s,lac,max_-q_s,glu_ correlation of the GI strain was a result of the reduced number of *lacI* copies.

In accordance with sugar uptake rates, productivity and cell lysis differed for the plasmid containing GI strain as well. Productivity was clearly reduced as a result of less T7 RNA polymerase expression from the lacUV5 promoter and cell lysis and leakiness were decreased as well, which we hypothesized could either be an effect of the lower specific lactose uptake rate or the lower amount of product in the periplasm of the cells.

### 3.3. Toxicity of High Lactose Uptake Rates

**Figure 4:**
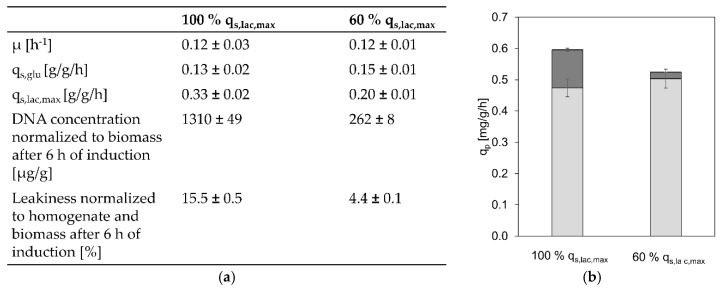
Physiology, leakiness, lysis and productivity at 100% and 60% q_s,lac,max_ (**a**) Comparison of physiological data as well as leakiness and lysis of a cultivation at a q_s,glu_ 0.14 g/g/h and different q_s,lac_ (100% q_s,lac,max_ and 60% q_s,lac,max_); (**b**) specific overall, intracellular (light grey), and extracellular (dark grey) product formation rates of cultivations at 100% q_s,lac,max_ and 60% q_s,lac,max_.

As including a pET-21 d (+) plasmid containing a *lacI* copy in the GI strain led to less cell lysis and leakiness, we were interested if the reason for this was the reduced productivity, putting less stress on the cells or the lower specific lactose uptake rate. For closer investigation we decided to perform two cultivations at the same q_s,glu_ (0.14 g/g/h) and at 100% q_s,lac,max_ as well as approximately 60% of q_s,lac,max_, as it has previously been shown that reducing q_s,lac,max_ to 57% still leads to comparable specific product formation rates (95.5% target protein) [20]. Results are shown in Figure 4.

As shown in Figure 4b, indeed a similar overall q_p_ was reached in both cultivations (88% of the q_p_ at 100% q_s,lac,max_ was still reached at 60% q_s,lac,max_). However, in the cultivation at 60% q_s,lac,max_ more product is found intracellularly and less in the cultivation supernatant. This is a result of less cell lysis and leakiness as shown in Figure 4a, indicating that not recombinant protein production but the high lactose uptake rates are toxic for the cells.

### 3.4. Comparison between Lactose Induction and IPTG Induction

**Figure 5:**
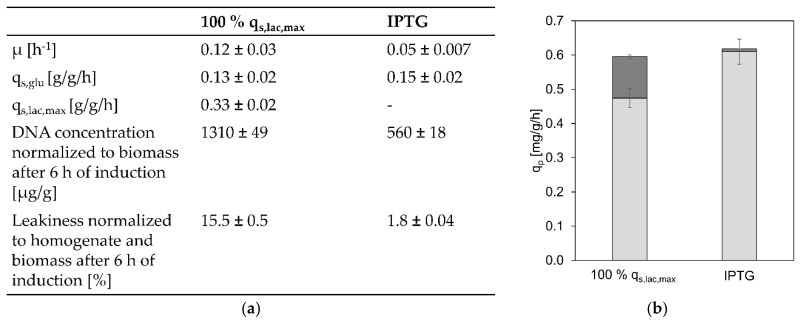
Physiology, leakiness, lysis, and productivity when inducing FabZ production by lactose or IPTG. (**a**) Comparison of physiological data as well as cell lysis and leakiness of the lactose induction cultivation at which the highest q_p_ was reached and an IPTG-induced cultivation conducted at the same q_s,glu_; (**b**) specific overall, intracellular (light grey), and extracellular (dark grey) product formation rates of the lactose-induced cultivation at which the highest q_p_ was reached and an IPTG-induced cultivation conducted at the same q_s,glu_.

For plasmid-based systems it has been reported that lactose induction provided the advantages of increased soluble product formation and cell fitness [19,20,21,22]. However, as the high specific lactose uptake rates of the GI strain clearly seemed toxic to the cells, we wanted to check if perhaps IPTG induction was beneficial for this strain. Therefore, we compared data obtained from the cultivation with lactose induction resulting in the highest q_p_ to an IPTG-induced cultivation that was conducted at a similar q_s,glu_. For comparability, both inducers were provided in excess. The physiological parameters as well as the specific product formation rates and the cell-lysis data are shown in Figure 5.

As shown in Figure 5, the overall q_p_ is similar in the lactose and the IPTG-induced process, however, the extracellular product formation is clearly reduced in the IPTG-induced process in comparison to lactose-induced cells. This most likely is a result of less cell lysis and leakiness in the IPTG-induced cultivation as shown in Figure 5a. All of this indicates that for a GI strain, with the GOI under control of the T7 promoter, lactose induction does not provide the same advantages which were previously discovered for plasmid-based systems. On the contrary, it seems to slightly reduce overall productivity and clearly diminishes cell viability compared to IPTG induction.

## 4. Conclusions

In this study we investigated the effects of lactose induction on an *E. coli* BL21 (DE3) expression system with a genome-integrated GOI. Previously, only plasmid-based expression systems had been subject to studies investigating lactose as an inducer for recombinant protein production [23,36,37]. In those studies, beneficial effects on productivity, cell fitness, viability, and soluble product formation had been reported [21,22,38,39,40].

Within this study we showed, for the first time, that an *E. coli* BL21 (DE3) expression system with a genome-integrated GOI showed specific maximum lactose uptake rates that were more than double as high compared to pET-plasmid based systems. We demonstrated that this difference is caused by the lack of a plasmid carrying additional copies of *lacI* and further showed that these unusually high lactose uptake rates of more than 0.4 g/g/h were toxic to the cells leading to increased cell lysis and product loss in the supernatant. Therefore, we conclude that previously discovered benefits of lactose induction are not applicable to strains with genome integrated GOIs under control of the T7 promoter.

## Supplementary Materials

The following are available online at https://www.mdpi.com/2306-5354/7/1/8/s1, Table S1: Physiological parameters of cultivations at q_s,lac,max_ at different q_s,glu_.
