# Supplementary material for: The Effects of Lactose Induction on a Plasmid-Free E. coli T7 Expression System
Source: Bioengineering (Basel). 2020 Jan 6;7(1):8. doi: 10.3390/bioengineering7010008 (PMC7175309; doi:10.3390/bioengineering7010008)
Supplement: Supplementary file 1 [file bioengineering-07-00008-s001.pdf]

bioengineering

Supplementary Material

Table S1. Physiological parameters of the strain at different $q_{s,glu}$ and the respective $q_{s,lac,max}$

$q_{s,glu}$ [g/g/h]	$q_{s,lac,max}$ [g/g/h]	μ [h^{-1}]
n.d.	0.17 ± 0.05	0.06 ± 0.02
0.19 ± 0.04	0.33 ± 0.05	0.22 ± 0.05
0.41 ± 0.07	0.40 ± 0.04	0.29 ± 0.07
0.80 ± 0.08	-	0.33 ± 0.07

© 2020 by the authors. Licensee MDPI, Basel, Switzerland. This article is an open access article distributed under the terms and conditions of the Creative Commons Attribution (CC BY) license (http://creativecommons.org/licenses/by/4.0/).
